# Acute skin toxicity of conventional fractionated versus hypofractionated radiotherapy in breast cancer patients receiving regional node irradiation: the real-life prospective multicenter HYPOBREAST cohort

**DOI:** 10.1186/s12885-022-10402-z

**Published:** 2022-12-16

**Authors:** Marie Bruand, Julia Salleron, Sébastien Guihard, Charles Marchand Crety, Xavier Liem, David Pasquier, Assia Lamrani-Ghaouti, Claire Charra-Brunaud, Didier Peiffert, Jean-Baptiste Clavier, Emmanuel Desandes, Jean-Christophe Faivre

**Affiliations:** 1 Academic Department of Radiation Therapy & Brachytherapy, Institut de Cancérologie de Lorraine – Unicancer, 6 avenue de Bourgogne - CS, 30 519 54519 Vandoeuvre-lès-Nancy cedex, France; 2 EA 4360 APEMAC, Université de Lorraine, Nancy, France; 3 Unité de biostatistiques, Institut de Cancérologie de Lorraine, 54519 Vandœuvre-lès-Nancy, France; 4 Service de Radiothérapie, ICANS - Institut de Cancérologie Strasbourg Europe, 67200 Strasbourg, France; 5 Service de Radiothérapie, Institut Jean Godinot, 51100 Reims, France; 6 Service de Radiothérapie, Centre Oscar Lambret, 59000 Lille, France; 7 RIStAL, UMR 9189, Université de Lille, 59000 Lille, France; 8 UNICANCER, R&D Department, 75013 Paris, France; 9 Service en Charge des Données de Santé, Institut de Cancérologie de Lorraine, 54519 Vandœuvre-lès-Nancy, France

**Keywords:** Breast neoplasms, Adjuvant radiotherapy, Radiation dose, Hypofractionation, Radiotherapy dose fractionation, Conformal radiotherapy, Radiotherapy, Intensity-modulated, Observational study, Prospective studies

## Abstract

**Background:**

Large-scale trials have shown that hypofractionated adjuvant breast radiotherapy was as effective in terms of survival and local control as conventional fractionated radiotherapy, and acute toxicity was reduced with hypofractionated radiotherapy. However, there is a lack of data about the toxicity of breast with regional nodal irradiation (RNI). The aim of this study was to assess the effect of fractionation on radiation-related acute skin toxicity in patients receiving RNI in addition to whole-breast or chest wall irradiation, using real-life data.

**Methods:**

We conducted a prospective, multicenter cohort study with systematic computerized data collection integrated into Mosaiq®. Three comprehensive cancer centers used a standardized form to prospectively collect patient characteristics, treatment characteristics and toxicity.

**Results:**

Between November 2016 and January 2022, 1727 patients were assessed; 1419 (82.2%) and 308 (17.8%) patients respectively received conventional fractionated and hypofractionated radiation therapy. Overall, the incidence of acute grade 2 or higher dermatitis was 28.4% (490 patients). Incidence was lower with hypofractionated than with conventional fractioned radiation therapy (odds ratio (OR) 0.34 [0.29;0.41]). Two prognostic factors were found to increase the risk of acute dermatitis, namely 3D (vs IMRT) and breast irradiation (vs chest wall).

**Conclusion:**

Using real-life data from unselected patients with regional nodal irradiation, our findings confirm the decreased risk of dermatitis previously reported with hypofractionated radiation therapy in clinical trials. Expansion of systematic data collection systems to include additional centers as well as dosimetric data is warranted to further evaluate the short- and long-term effects of fractionation in real life.

**Supplementary Information:**

The online version contains supplementary material available at 10.1186/s12885-022-10402-z.

## Introduction

The crucial role of adjuvant radiotherapy on local control and survival in breast cancer has previously been validated [1]. Breast irradiation after conservative treatment has therefore been standard of care for many years. Historically, the treatment regimen consists of 25 sessions, over 5 weeks, which may be supplemented by a “boost” to the surgical tumor bed [2]. This high number of sessions is associated with significant treatment costs [3] and involves constraints related to daily transport. Furthermore, it may lead to fatigue and deterioration of patients’ quality of life, and contributes to the saturation of radiotherapy departments. These drawbacks prompted randomized trials to compare conventional fractionated vs hypofractionated breast radiotherapy [4–7]. These trials demonstrated comparable efficacy, and have therefore led to changes in radiotherapy practices in the last few years. In addition, recent studies have shown lower incidence and severity of skin toxicity with hypofractionated treatment regimens, in selected patients in whom the breast alone was irradiated [8, 9]. However, most patients included in these trials received radiotherapy that did not include regional lymph nodes.

To date, few studies and reviews are available comparing the occurrence of acute toxicity according to fractionation, when the irradiation includes regional lymph nodes. These studies did not report any increase in skin toxicity with RNI, but their level of evidence remains insufficient [10, 11].

Despite the limited data concerning the safety of breast and regional node irradiation using a hypofractionated schedule, a shift in practice has been observed in recent years in crisis contexts (staff shortages, COVID-19 pandemic). Recently, the European Society for Radiotherapy and Oncology Advisory Committee in Radiation Oncology Practice issued a consensus statement, recommending use of hypofractionation for RNI [12]. There is therefore a need to evaluate these practices. The aim of this study was to assess the effect of fractionation on radiation-related acute skin toxicity in patients receiving RNI in addition to whole-breast or chest wall irradiation, using real-life multicentre data.

## Methods

### Setting, design and participants

The HYPOBREAST study (HYPOfractionation in BREAST radiation therapy) was an observational, prospective, multicenter cohort study, involving three French comprehensive cancer centers between November 2016 and January 2022.

Female patients over 18 years old, with localized breast carcinoma, receiving RNI were included. RNI was defined as internal mammary and supraclavicular irradiation, with or without axillary lymph nodes irradiation. The exclusion criteria were male patients, whole-breast or chest wall alone with no regional node irradiation, extreme hypofractionation and missing data for acute toxicity outcomes.

### Data collection and follow-up

Demographic data, medical history, clinical characteristics, and tumor characteristics were collected at the first medical visit. Radiotherapy characteristics were collected at the time of prescription of the radiotherapy treatment. Acute toxicity data were collected at weekly follow-up visits during treatment. No toxicity evaluation occurred within 3 months after the end of radiotherapy. All data were collected by systematic data recording on a standardized, computerized form using the MOSAIQ® software (Elekta AB, Stockholm, Sweden).

Demographic data collected were age at the start of radiotherapy and sex. Regarding medical history, we recorded: history of diabetes, menopausal status, active smoking, and body mass index (BMI). Tumor characteristics were TNM classification (American Joint Commission on Cancer 8th edition), hormone and HER2 status, and tumor topography. Radiation therapy characteristics recorded were: CTVs, PTVs treated, total dose, dose per fraction, number of fractions, technique (intensity-modulated radiation therapy (IMRT) or 3D conventional), use of electrons, and energy (MV). Toxicity data collected were only acute skin toxicity.

### Treatment regimen

External beam radiotherapy was delivered with a conventional 3D conformal or IMRT technique, with 6–20 MV X-ray beam energy (separated into standard energy for 6 MV photons, and high energy if 15 MV or higher), with or without use of electrons mixed with photons, with or without an additional dose to the tumor bed (“boost”). Hypofractionation was defined as fractions exceeding 2.2 Gray per fraction, and extreme fractionation as fractions exceeding 6 Gray per fraction.

### Outcomes

The primary outcome was acute skin toxicity occurring during treatment, defined as the grade of dermatitis according to the Common Terminology Criteria for Adverse Events (CTCAE) version 4. We grouped grades 0 and 1 together, representing no or low toxicity, and grades 2 and 3 together, representing toxicity requiring treatment.

### Statistical analysis

Categorical variables are described as number and percentage, and quantitative variables as mean and standard deviation if normally distributed, or median and interquartile range if non-normally distributed. Normality was tested with the Kolmogorov-Smirnov test. Incidence of toxicity is described in the conventional fractionated (CF) and hypofractionated (HF) groups as percentage with 95% confidence interval (95%CI). Demographic data, medical history, clinical and tumor characteristics were compared between CF and HF groups by calculating absolute standardized differences (S_diff_) and *p* values, using Student’s t test for quantitative variables and the Chi-Square test for categorical variables.

The impact of fractionation on acute toxicity was assessed using bivariate logistic regression. To adjust the results for potential selection bias (i.e. the choice of fractionation could be made based on patient characteristics), bivariate analyses were adjusted for imbalanced parameters between the two groups of fractionation. The imbalance was considered a negligible difference if S_diff_ was < 10% [13]. For the purposes of adjustment, a propensity score (PS) was used, based on the Inverse Probability of Treatment Weighting (IPTW) method [13, 14]. The weight assigned to individuals was 1/PS in the HF group, and 1/(1-PS) in the NF group. The PS was calculated using a logistic regression model including relevant variables associated (i.e. |S_diff_| > 10%) with toxicity and fractionation (potential confounders) [13]. Details of the IPTW method are provided in the Supplementary Material. Finally, the effect of fractionation on acute toxicity was estimated by weighted logistic regression using the IPTW method. Sensitivity analysis using the same methods was performed in the subgroup of patients with available data for BMI and smoking status.

An exploratory subgroup analysis was performed to determine whether the effect of hypofractionation was different according to the initial characteristics of the patients. For each baseline variable, we estimated an odds ratio for toxicity of the HF group compared to the CF group, in each stratum of the variable, and tested for a significant interaction.

The predictive factors of acute toxicity in hypofractionated population were assessed by bivariate logistic regression. All parameters with a *p*-value < 0.20 in bivariate analysis were included in a full multivariate logistic regression. To respect the principle of parsimony, the full model was simplified using backward selection. Only the results of the final multivariate model are presented.

To describe the progression in the use of hypofractionation over time, a new variable representing time standardization over 3-month periods was considered. The percentage of HF treatment over time was plotted. The percentage of hypofractionation before and after the start of COVID-19 pandemic in France was compared using the Chi-Square test.

All analyses were performed using SAS version 9.4 (SAS Institute Inc., Cary, NC, USA).

## Results

### Participants

**Fig. 1:**
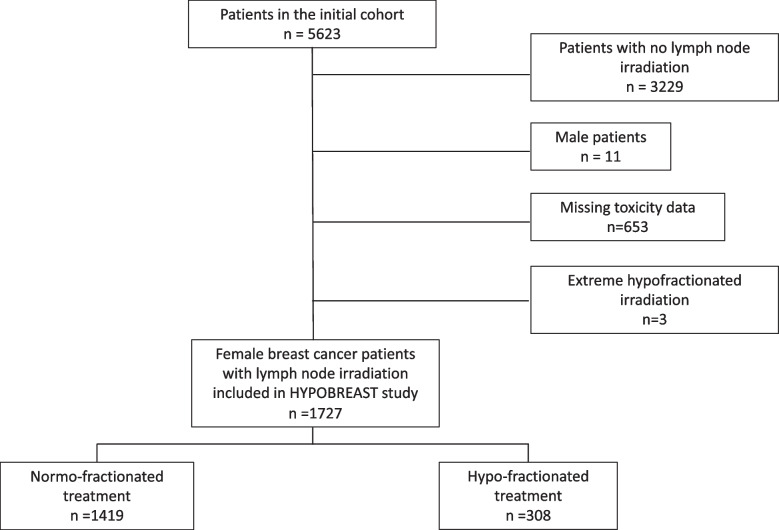
Flow chart of patient selection

The initial cohort enrolled 5623 adult patients treated with radiation therapy as adjuvant treatment for breast cancer from November 2016 to January 2022. We excluded 3229 patients who had no RNI, and a further 667 who met other exclusion criteria. The flowchart of patient selection is shown in Fig. 1. A final total of 1727 female patients who received RNI were included (Fig. 1).

### Characteristics at inclusion

Patient, tumor and treatment characteristics in overall population and according to the 2 fractionation groups are presented in Table 1. The mean age of the population was 58.8 ± 13.8 years. Overall, 17.8% of patients (*n* = 308) received hypofractionated treatment. A total of 55.9% received breast irradiation, 44.1% received chest wall irradiation and 46.0% received a boost to the surgical tumor bed. Treatment was performed with IMRT in 45.2% of cases, with the use of electrons mixed with photons in 30.8% of cases, and the use of high energy photons in 51.8%. Patients in the HF group were older (*p* < .0001), more likely to receive treatment to the breast (vs chest wall) (*p* < .0001), more likely to be treated with IMRT (vs 3D conformal technique) (p < .0001), less likely to receive a boost (vs no boost) (*p* = 0.001), or to be treated with high energy photons (vs standard energy) (p < .0001) or electrons (vs photons only) (p < .0001). The description of imbalanced parameters with their |S_diff_| before and after application of the IPTW method is presented in the Supplementary Material.

**Table 1:** Patient, tumor and treatment characteristics between conventional fractionation (CF) and hypofractionation (HF) groups

	All patients (*n* = 1727)	Conventional fractionated (*n* = 1419)	Hypofractionated (*n* = 308)	*P* value
Age (years), mean (SD) (n_T_ = 1727)	58.8 (13.8)	57.8 (13.5)	63.0 (14.5)	< 0.0001
BMI (kg/m^2^), median [IQR] (n_T_ = 1017)	26.6 [23.2; 30.9]	26.6 [23.2; 30.9]	26.4 [22.7; 30.4]	0.41
Active Smokers (n_T_ = 1478)	207 (14.0)	189 (15.2)	18 (7.6)	0.002
Diabetes (n_T_ = 635)	79 (12.4)	68 (12.3)	11 (13.1)	0.85
Menopausal status (n_T_ = 536)				
Menopause	464 (86.6)	406 (85.1)	58 (98.3)	0.017
Perimenopause	26 (4.8)	25 (5.2)	1 (1.7)	
No Menopause	46 (8.6)	46 (9.6)	0	
Breast side (Right) (n_T_ = 1668)	812 (48.7)	687 (49.4)	125 (45.0)	< 0.0001
Quadrant (n_T_ = 1343)				
Upper-Outer	561 (41.8)	478 (41.1)	83 (44.1)	
Overlapping lesion of breast	295 (22.0)	256 (22.2)	39 (20.7)	< 0.0001
Upper-Inner	153 (11.4)	128 (11.1)	24 (12.8)	
Central portion of breast	140 (10.4)	115 (10.0)	25 (13.3)	
Lower-Outer	108 (8.0)	96 (8.3)	12 (6.4)	
Lower-Inner	72 (5.4)	69 (6.0)	3 (1.6)	
Axillary tail of breast	14 (1.0)	12 (1.0)	2 (1.1)	
Molecular subtype (n_T_ = 1163)				
Triple negative	155 (13.3)	135 (13.4)	20 (13.2)	0.016
HR- HER2+	95 (8.2)	85 (8.4)	10 (6.6)	
HR+ HER2+	132 (11.3)	106 (10.5)	26 (17.1)	
HR+ HER2-	781 (67.2)	685 (67.8)	96 (63.2)	
Grade (n_T_ = 1274)				0.1011
1	187 (14.7)	150 (13.8)	37 (19.8)	
2	607 (47.6)	524 (48.2)	83 (44.4)	
3	480 (37.7)	413 (38.0)	67 (35.8)	
pT stage (n_T_ = 1333)				
0	136 (10.2)	120 (10.5)	16 (8.3)	0.1260
1	458 (34.4)	402 (35.2)	56 (29.2)	
2	547 (41.0)	454 (39.8)	93 (48.4)	
3	153 (11.5)	134 (11.7)	19 (9.9)	
4	39 (2.9)	31 (2.7)	8 (4.2)	
pN stage (n_T_ = 1359)				
pN+	1021 (75.1)	865 (74.3)	156 (80.0)	0.0879
Breast/Chest Wall (n_T_ = 1727)				
Breast	966 (55.9)	746 (52.6)	220 (71.4)	< 0.0001
Chest wall	761 (44.1)	673 (47.4)	88 (28.6)	
Axillary irradiation (n_T_ = 1727)	87 (5.0)	63 (4.4)	24 (7.8)	0.01
Technique (n_T_ = 1727)				
IMRT	781 (45.2)	523 (36.9)	258 (83.8)	< 0.0001
3D	946 (54.8)	896 (63.1)	50 (16.2)	
Use of electrons (n_T_ = 1727)	531 (30.8)	519 (36.6)	12 (3.9)	< 0.0001
Tumor bed boost (n_T_ = 1727)	795 (46.0)	679 (47.9)	116 (37.7)	0.001
Photon Energy (n_T_ = 1727)				
Standard	833 (48.2)	574 (40.5)	259 (84.1)	< 0.0001
High	894 (51.8)	845 (59.5)	49 (15.9)	
Center (n_T_ = 1727)				< 0.0001
Center 1	1035 (59.9)	900 (63.4)	135 (43.8)	
Center 2	574 (33.2)	467 (32.9)	107 (34.7)	
Center 3	118 (6.8)	52 (3.7)	66 (21.4)	

*BMI* Body Mass Index, *HER2* Human Epidermal Growth factor 2, *HR* Hormone Receptors, *IMRT* Intensity Modulated Radiation Therapy, *IQR* Interquartile Range, *n*_*T*_ number of patients with available information, *pN* pathological Node (TNM classification), *pT* pathological Tumor, *SD* standard deviation, *3D* 3D conformal. Results presented as n/n_T_ (%) unless otherwise specified. Percentages may not total 100 because of rounding

### Toxicity

A total of 490 (28.4%) patients presented radiation-induced grade 2 or higher acute dermatitis during treatment. The baseline characteristics by toxicity group are presented in Supplementary Table S1.

Grade 2 or higher dermatitis was reported in 10.7% (95%CI [7.3;14.2]) in the HF group and 32.2% (95%CI [29.8;34.6]) in the CF group (OR 0.25, 95%CI [0.17;0.37], *p* < 0.0001). After considering the imbalance in initial characteristics between groups (see Supplementary Table S1 and S2 and Supplementary Fig. 1), the difference in the rate of grade ≥ 2 dermatitis remained statistically significant (OR 0.34, 95%CI [0.29;0.41]). Among the 1727 patients, 752 (43.5%) had complete data available regarding smoking status and BMI. Sensitivity analysis performed in these patients confirmed the results observed in the overall population (OR 0.10, 95%CI [0.07;0.14]). Details are presented in Supplementary Tables S3 to S7 and Supplementary Fig. 2.

**Fig. 2:**
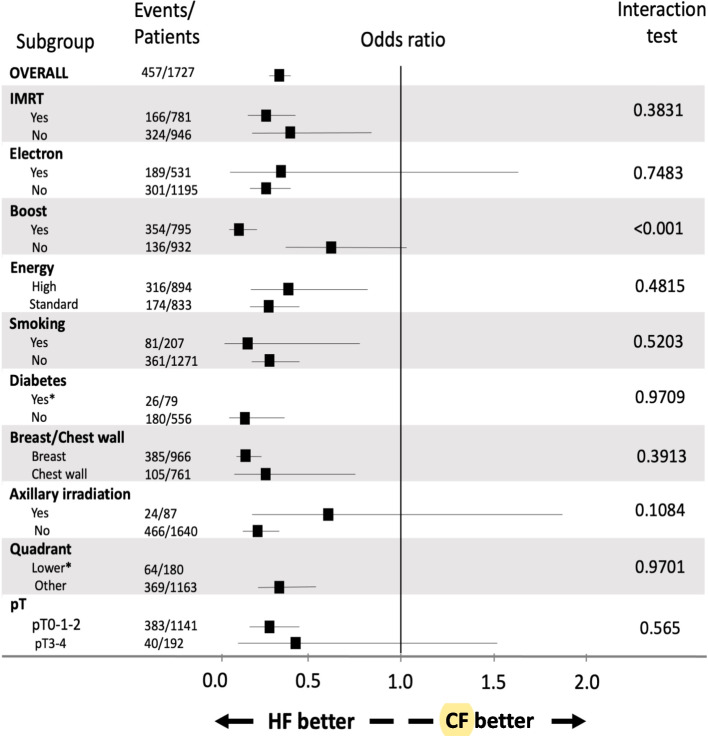
Effects of fractionation on acute skin toxicity across subgroups of baseline characteristics. *Legend:* HF Hypofractionated, CF Conventional fractionated. Events represents the number of acute skin toxicity. The p-value is from the test statistic for testing the interaction between fractionation and any subgroup parameters. *No event in hypofractionated group

The effect of HF vs CF treatment within each strata of the baseline variables is presented in Fig. 2. The impact of HF treatment was similar across all subgroup analyses, and the only significant interaction observed was between fractionation and presence/absence of boost (*p* < 0.001), whereby patients who received a boost developed significantly less acute toxicity in the HF group compared to the CF group (OR 0.12, 95%CI [0.07;0.23]) whereas there was no significant difference in toxicity rates between fractionation groups in patients who received no boost (OR 0.63, 95%CI [0.38;1.03]).

#### Predictive factors of toxicity

Table 2 summarizes the significant predictors of radiation-induced dermatitis in bivariate and multivariate analyses. In the final multivariate analysis, 2 factors were found to be independently associated with the risk of dermatitis: patients with breast irradiation were more likely to develop radiation-induced dermatitis during treatment than patients with chest wall irradiation (OR 5.74, 95%CI [1.64;20.05]), while patients treated with the IMRT technique were less likely to develop dermatitis than patients treated with the 3D conformal technique (OR 0.33, 95%CI [0.14;0.80]).

**Table 2:** Predictive factors of toxicity in the population receiving hypofractionated therapy (*N* = 308)

	Bivariate analysis		Final multivariate model*	
	OR, 95% CI	p	OR, 95% CI	p
Breast (vs chest wall) ^∆^	4.47 [1.33;15.06]	0.02	5.74 [1.64;20.04]	0.006
Technique IMRT (vs 3D) ^∆^	0.47 [0.20;1.08]	0.07	0.30 [0.14;0.80]	0.014
Axillary irradiation ^∆^	2.41 [0.83;6.94]	0.10		
Energy (high vs standard) ^∆^	2.20 [0.96;5.08]	0.06		
Electrons	0.47 [0.36;8.13]	0.50		
Tumor bed boost	1.08 [0.52;2.27]	0.83		
Smoking	0.92 [0.20;4.25]	0.92		

*OR* odds ratio, *CI* confidence interval, *IMRT* Intensity Modulated Radiation Therapy, *3D* 3D conformal

^∆^ Included in the full multivariate analysis

*Final model after backward selection

### Use of hypofractionation over time

The proportion of irradiation performed using HF was 9.3% before the start of the COVID-19 pandemic in France in February 2020, and rose to 30.4% in January 2022 (*p* < 0.0001). A histogram of the percentage of NF and HF treatments over time is presented in Supplementary Fig. 3.

## Discussion

This study shows a major decrease in radiation-induced dermatitis during treatment with HF compared to CF radiation therapy, with a threefold reduction in the risk of dermatitis. The analyse of effect of HF vs CF treatment within each strata of the baseline variables, revealed a decrease in dermatitis in HF compared to CF group, regardless of treatment site (breast or chest wall), treatment technique (IMRT or conformal), energy type, smoking status, when the treatment included a boost, in the absence of axillary irradiation, and with use of electrons. Conversely, there was no significant difference between HF and CF radiation therapy, when treatment did not include a boost, did not use electrons, or in case of axillary irradiation. The investigation of predictive factors of toxicity in the HF group was exploratory, given the small number of patients, but nonetheless revealed a significant relationship between the irradiation technique and irradiation of the chest wall or breast. The percentage of HF radiotherapy in patients with RNI in our cohort increased between early 2016 and the January 2022. The increase was not linear, and an abrupt change was visible that coincided with the start of the COVID-19 pandemic, rising from around 10% to more than 30% in a few months.

Most studies reporting acute toxicity involved patients who did not have RNI. In spite of this difference, the incidence of toxicity reported in these studies was broadly comparable to that observed in the present HYPOBREAST study. Indeed, the incidence of grade 2 or higher acute dermatitis in patients treated with a HF regimen varied from 5 to 27% in most studies, which is consistent with the 10.7% incidence found in our study. In all these studies, the comparison between the HF and CF groups showed a 2 to 3-fold decrease in acute skin toxicity with hypofractionation, which is also comparable to our results [8, 9, 15–17]. Arsenault et al. went further by also analyzing the duration of acute skin injury, and showed that both the peak and the duration of toxicity were reduced by HF compared to CF [8]. The Beijing trial recruited exclusively patients treated on the chest wall with RNI. This trial analyzed skin toxicities by grouping grade 1 and 2, which makes it difficult to compare with our data; however, they found no significant difference between the CF and HF groups [18]. A recent review and meta-analysis of studies that included post-mastectomy irradiation also failed to show any significant difference between CF and HF groups [19]. The START B trial enrolled 7.4% of patients with RNI, and reported no major skin toxicity in this population [4].

Regarding the predictive factors of toxicity in patients treated with HF therapy, no study to date has investigated the predictors of acute dermatitis in this specific population. However, some studies have assessed the risk factors for toxicity in a NF population, and have reported that IMRT is a technique that can reduce skin toxicity, which is consistent with our findings [20, 21]. On the other hand, we describe a significant relationship between acute dermatitis and irradiation of the chest wall or breast, with lower toxicity when the chest wall is irradiated. It has been reported that severe acute cutaneous toxicities are more often observed in the axillary and infra-mammary folds, via a self-bolus effect [22]. Self-bolus effect is the removal of skin sparing effects of megavoltage radiation beams due to build-up of skin folds. It therefore seems logical that fewer grade 2 or higher acute skin toxicity events would be observed in patients receiving post-mastectomy irradiation.

In our study, the use of hypofractionation was different between centers. We therefore included the center in the propensity score, to limit the potential confounding bias that this difference might have generated in our analysis. This difference highlights the heterogeneity in the use of hypofractionated radiotherapy, which has been previously described in the literature. Indeed, Prades et al. attempted to understand the variation in hypofractionation use and the clinical and organizational factors influencing the OR decision [23]. They described clinical factors such as age, indication for chemotherapy, left side, indication for NIR, large breast, chest irradiation, indication for boost, or certain histological subtypes. The authors conclude that the clinical factors cited have little basis in scientific evidence and that factors related to the management of radiotherapy services play a major role. A more recent study, by Ratosa et al., sought to describe the fractionation preferences of radiation oncologists across Europe. Only 28.7% preferred a hypofractionated regimen when irradiating the lymph nodes, while 29.6% preferred a hypofractionated regimen when irradiating the chest wall after mastectomy [24]. The authors also described the reasons influencing the decision to use hypofractionation. The most frequently cited reasons were young age, lymph node irradiation, post-mastectomy indications and breast reconstruction, especially as these are subgroups of patients less represented in the literature. To a lesser extent, organizational aspects and financial issues also had an influence. These study, as well as our cohort, confirms the difficulties of implementing hypofractionated regimens in clinical routine. Indeed, although hypofractionation has many advantages such as patient convenience, accessibility, reduction of waiting lists, better use of limited resources, cost effectiveness, it is still not widely used despite a high-level concerning effectiveness and safety of this approach. As pointed out by Ratosa et al., one of the disadvantages of hypofractionation in some countries is the financial and reimbursement issue. An ESTRO-HERO analysis of reimbursement in Europe, by Lievens et al., highlighted the variability of reimbursement for radiotherapy treatments and the existence of systems that are not adapted to the recent evolution of radiotherapy, such as hypofractionation, and therefore the need to discuss new reimbursement strategies that would allow radiotherapy department to follow evidence-based treatment without being financially disadvantaged [25].

The absence of data concerning breast volume could be a source of potential bias. Indeed, breast size is a risk factor for dermatitis, as previously described in patients treated by conventional fractionated radiotherapy [26, 27]. We cannot exclude the possibility that the breast volume modifies the attitude of the radiation oncologist, and therefore, that patients with a larger breast volume may be more represented in the normofractionated population, which could artificially increase the difference between the 2 groups. We conducted a sensitivity analysis on a sub-cohort with more data (BMI and smoking status). Patients in the sub cohort were less likely to receive axillary irradiation, more likely to receive a boost and to be treated electrons. These differences may affect the generalizability of the results of the sensitivity analysis to the total cohort. However, axillary irradiation did not seem to be related with acute toxicity in our study. Electrons use and boost were more frequent in the sub cohort and could therefore cause greater toxicity in the sub cohort. Despite this, the toxicity reduction with HF, compared to CF radiotherapy, was even more important in the sub cohort. Finally, this sensitivity analysis showed that taking smoking status or BMI into account did not alter the results, and therefore the strength of association is such that even if possible confounding bias existed that was not taken into account, it would not change the final result. In addition, we know that the use of high-energy photons occurs when breast volume is high, and we have shown that hypofractionation was less toxic even when high-energy photons were used. In their study in a population receiving hypofractionated radiotherapy, Janssen et al. did not find any link between breast- or boost-volume, and acute and late toxicity [15]. Corbin et al. compared hypofractionated with conventional fractionated radiotherapy in a large-breasted population [28], and concluded that, in obese and large-breasted populations, there was no increase in acute skin toxicity with the use of hypofractionation. These studies therefore reinforce our conclusion that hypofractionation reduces acute cutaneous toxicities in any population.

Despite clinical trials proving the efficacy and safety of hypofractionated schedules, less than half of patients are treated with hypofractionated schedules. It seems necessary to promote this form of treatment, both for the comfort of patients and to enhance the accessibility of radiotherapy treatments by reducing costs [29, 30]. Moreover, our study demonstrates the feasibility of using real-life data from systematic computerized data collection to conduct large-scale Phase IV studies. Indeed, real-life studies are essential to provide effectiveness data, which complement the efficacy data generated by randomized studies. These two types of study are complementary, because they provide different types of information to clinicians. The development of systematic data collection should be encouraged to collect more population-based data, and should also be extended to include dosimetric data, with a view to strengthening the methodology of real-life studies, and better informing clinicians for their daily practice.

## Conclusion

Using real-life data from unselected patients with regional nodal irradiation, our findings confirm the decreased risk of dermatitis previously reported with hypofractionated radiation therapy in clinical trials. Expansion of systematic data collection systems to include additional centers as well as dosimetric data is warranted to further evaluate the short- and long-term effects of fractionation in real life.

## Supplementary Information

**Additional file 1.**

## Data Availability

The data are not publicly available due to them containing information that could compromise research participant privacy or consent but are available from the corresponding author on reasonable request.

## Abbreviations

BMI: Body Mass Index
CF: Conventional fractionated
HF: hypofractionated
IMRT: Intensity Modulated Radiation Therapy
IPTW: Inverse Ponderation Treatment Weighting
OR: Odds ratio
PS: Propensity Score
RNI: Regional Node Irradiation
S_diff_: Standardized Difference
