# Supplementary material for: Acute skin toxicity of conventional fractionated versus hypofractionated radiotherapy in breast cancer patients receiving regional node irradiation: the real-life prospective multicenter HYPOBREAST cohort
Source: BMC Cancer. 2022 Dec 16;22:1318. doi: 10.1186/s12885-022-10402-z (PMC9755801; doi:10.1186/s12885-022-10402-z)
Supplement: Supplementary file 1 — Additional file 1. [file 12885_2022_10402_MOESM1_ESM.docx]

Supplementary data

*Propensity score construction*

We first investigated to identify potential confounders. This analysis was limited to selected relevant baseline variables for which there was clinical suspicion, or that had previously been described in the literature. For this purpose, we calculated standardized differences for baseline variables between HF and NF groups and between acute toxicity and no acute toxicity groups. The imbalance was considered as a negligible difference if the |S_diff_| was less than 10%. We then calculated a propensity score (PS) using a multivariate logistic regression model including selected variables associated (|S_diff_|>10%) with toxicity and fractionation (potential confounders).

To ensure that the model used would render the distribution of baseline covariates comparable between the HF and NF groups, we calculated |S_diff_| after IPTW. Moreover, to assess the quality of the PS, we checked that the common support was large enough. The remaining imbalance (|S_diff_|>10% and p value <0.05) was handled as a dependent covariate in the weighted logistic regression.

*Detailed results in the overall population (n=1727)*

Baseline characteristics in the 2 toxicity groups (acute toxicity and no acute toxicity), along with their |S_diff_| are presented in Supplementary Table 1. Differences between the 2 toxicity groups identified were: breast or chest wall irradiation (|S_diff_|=69.2%), Technique (|S_diff_|=32.5%), electron use (|S_diff_|=23.3), Boost (|S_diff_|=78.9%), Energy (|S_diff_|= 36.3) and Centers 1 (|S_diff_|=19.7) and 3 (|S_diff_|=36.7).

Potential confounder distributions in the 2 fractionation groups, along with their |S_diff_| are presented in Supplementary Table 2 and Supplementary Figure 1, after weighting. Covariates identified as potential confounders and used in the logistic regression model to build the propensity score were: age, breast/chest wall, technique, electron use, energy, boost, center.

After weighting on the PS, one variable remained significantly different between groups, namely the use of electrons (|S_diff_|=18.5%) (Supplementary Figure 1), and was included as a dependent covariate in the weighted logistic regression. Despite the persistence of an imbalance in one variable, the propensity score was of good quality with a significant reduction in bias for all variables, and balance was achieved between groups for all variables except one after weighting, and common support was large (0.0038; 0.93).

*Detailed results in the population with available data for body mass index (BMI) and smoking status (n= 752)*

Since BMI and active smoking were not included in previous analyses, due to high rates of missing data, we performed a sensitivity analysis among patients with complete data available for BMI and smoking. The purpose of this sensitivity analysis was to confirm the stability of the results when these factors are considered.

A total of 752 (43.5%) patients had complete data for smoking status and BMI. Initial characteristics of this population are presented in Supplementary Table 3. The mean age of the population was 59.2 years; 11.3% received HF treatment. 55.2% of the patients received breast irradiation, 44.8% received chest wall irradiation and 55.7% received a boost to the tumor bed. Radiotherapy was performed with IMRT in 42.7% of cases, with the use of electrons in 52.4% of cases, and the use of high energy photons in 52.4% of cases.

Baseline and treatment characteristics according to the 2 fractionation groups are presented in Supplementary Table 4. Patients in the HF group were older (p<.0001), less often smokers (p=0.002), more likely to be treated with IMRT (p<.0001), or to receive a boost (p=0.03), and less likely to be treated with high energy photons (p<.0001) or electrons (p<.0001).

A total of 254 (33.8%) patients presented radiation-induced grade 2 or higher dermatitis during treatment: 37.3% (95% CI [33.6;41.1]) of patients treated with NF radiation therapy presented a grade 2 or higher dermatitis, vs only 5.88 % (95%CI [1.9;13.2]) in HF group. Baseline characteristics according to toxicity group are presented in Supplementary Table 5. Differences between the 2 toxicity groups identified were: age (|S_diff_|17.1%), BMI (|S_diff_|=34.4%), smoking status (|S_diff_|=17.6%), pT stage (|S_diff_|=38.2%), breast or chest wall irradiation (|S_diff_|=79.9%), technique (|S_diff_|=16.3%), electron use (|S_diff_|=13%), boost (|S_diff_|=73.4%), and energy (|S_diff_|=23.9%).

Potential confounders in the 2 fractionation groups, along with their |S_diff_| are presented in Supplementary Table 6 after weighting. Covariates identified as potential confounders and used in the logistic regression model to build the propensity score were: age, smoking status, breast/chest wall, technique, electron use, energy and boost.

After weighting on the PS, three variables remained significantly different between groups: smoking status (|S_diff_|= 21.3%), BMI (|S_diff_|= 12.9%) and boost (|S_diff_|= 12.7%) (Supplementary Figure 2). The difference in median BMI was not clinically relevant because it was only 1.75 kg/m² and both groups were in the same weight class. Smoking status and boost were handled as dependent covariates in the weighted logistic regression

Results of the sensitivity analyses along with adjustment variables are presented in Supplementary Table 7.

After adjustment, patients treated with HF radiation therapy had less dermatitis than patients treated with NF radiation therapy (OR 0.10, 95%CI [0.07;0.14]), confirming results of the principal analysis.

Supplementary Table S1. Patient, tumor and treatment characteristics distribution according to the occurrence of grade 2 or higher toxicity

|  | Acute toxicity (n=490) | No acute toxicity (n=1237) | \|Sdiff\| % |
| --- | --- | --- | --- |
| Age (years), mean (SD) (n_T_ =1727) | 57.8 (12.7) | 59.13(14.2) | 9.5 |
| BMI (kg/m²), median) [IQR] (n_T_ =1017) | 26.1 [22.7 ; 30.3] | 28.0 [24.1 ; 32.3] | 30.7 |
| Active Smokers (n_T_ =1478) | 81 (18.3) | 126 (12.2) | 17.21 |
| Diabetes (n_T_ =635) | 26 (12.6) | 53 (12.4) | 0.8 |
| Quadrant (n_T_ =1343) |  |  |  |
| Lower Quadrant | 64 (14.8) | 116 (12.7) |  |
| Other | 369 (85.2) | 794 (87.3) | 5.9 |
| pT stage (n_T_ =1333) |  |  |  |
| 0-1-2 | 383 (90.5) | 758 (83.3) | 7.2 |
| 3-4 | 40 (9.5) | 152 (16.7) |  |
| pN stage (n_T_ =1359) |  |  |  |
| pN+ | 320 (75.1) | 701 (75.1) | 0.02 |
| Breast/Chest Wall (n_T_ =1727) |  |  |  |
| Breast | 385 (78.6) | 581 (47.0) | 69.2 |
| Chest wall | 105 (21.4) | 656 (53.0) |  |
| Axillary irradiation (n_T_ =1727) | 24 (4.90) | 63 (5.09) | 0.9 |
| Technique (n_T_ =1727) |  |  |  |
| IMRT | 166 (33.9) | 615 (49.7) | 32.5 |
| 3D | 324 (66.1) | 622 (50.3) |  |
| Electrons (n_T_ =1727) | 189 (38.6) | 342 (27.7) | 23.3 |
| Boost (n_T_ =1727) | 354 (72.5) | 441 (35.7) | 78.9 |
| Photon Energy (n_T_ =1727) |  |  |  |
| Standard | 174 (35.5) | 659 (53.3) | 36.3 |
| High | 316 (64.5) | 578 (46.7) |  |
| Center (n_T_ =1727) |  |  |  |
| Center 1 | 327 (66.7) | 708 (57.2) | 19.7 |
| Center 2 | 159 (32.5) | 415 (33.6) | 2.3 |
| Center 3 | 4 (0.8) | 114 (9.2) | 36.7 |
| Hypofractionation (n_T_ =1727) |  |  |  |
| Yes | 33 (6.7) | 275 (22.2) |  |
| No | 457 (93.3) | 962 (77.8) |  |

BMI: Body Mass Index, IMRT: Intensity Modulated Radiation Therapy, IQR: InterQuartil Range, n_T_: number of patients with available information, pN: pathological Node (TNM classification), pT: pathological Tumor, S_diff_: Standardized Difference. SD: standard deviation, 3D: 3D conformal.

Results presented as n (%) unless otherwise specified. Percentages may not total 100 because of rounding.

Supplementary Table S2. Potential confounders before and after propensity score weighting

|  | Before weighting | | | After weighting | | | | |
| --- | --- | --- | --- | --- | --- | --- | --- | --- |
|  | Normo-fractionated (n=1419) | Hypo-fractionated  (n=308) | \|Sdiff\|  % | Normo-fractionated  (n=1414) | Hypo-fractionated  (n=307) | \|Sdiff\|  % | % bias reduction | p value |
| Age (years), mean (SD) | 57.8 (13.5) | 63.0 (14.5) | 37.5 | 59.3 (15.1) | 59.4 (38.1) | 0.6 | 98.3 | 0.90 |
| Breast/chest wall |  |  |  |  |  |  |  |  |
| Breast | 52.6 (746) | 71.4 (220) | 40.1 | 56.2 | 57.1 | 1.9 | 95.3 | 0.60 |
| Chest wall | 47.4 (673) | 28.6 (88) |  | 43.8 | 42.9 |  |  |  |
| Technique |  |  |  |  |  |  |  |  |
| IMRT w/o elec | 36.9 (523) | 83.8 (258) | 109.1 | 46.0 | 49.3 | 7.6 | 93.0 | 0.06 |
| 3D w/o elec | 26.6 (377) | 12.3 (38) | 36.4 | 23.8 | 27.3 | 9.1 | 75.1 | 0.02 |
| electrons | 36.6 (519) | 3.9 (12) | 89.0 | 30.2 | 23.4 | 18.5 | 79.2 | <0.0001 |
| Boost | 47.9 (679) | 37.7 (116) | 20.5 | 45.2 | 47.2 | 4.1 | 79.9 | 0.24 |
| Energy |  |  |  |  |  |  |  |  |
| Standard | 40.5 (574) | 84.1 (259) | 100.1 | 48.9 | 50.2 | 2.9 | 97.2 | 0.47 |
| High | 59.5 (845) | 15.9 (49) |  | 51.0 | 49.8 |  |  |  |
| Center |  |  |  |  |  |  |  |  |
| Center 1 | 63.4 (900) | 43.8 (135) | 39.7 | 59.7 | 57.6 | 4.2 | 89.4 | 0.26 |
| Center 2 | 32.9 (467) | 34.7 (107) | 4.1 | 32.2 | 34.8 | 5.5 | 0 |  |
| Center 3 | 3.7 (52) | 21.4 (66) | 55.0 | 8.1 | 7.6 | 1.8 | 96.8 |  |

IMRT w/o elec : Intensity Modulated Radiation Therapy without use of electrons, 3D w/o elec : 3D conformal technique without use of electrons, S_diff_: Standardized Difference, SD: standard deviation.

Results presented as % (n) unless otherwise specified. Percentages may not total 100 because of rounding.

Supplementary Table S3. Patient, tumor and treatment characteristics in patients with full data available for body mass index and smoking status

|  | All patients (n=752) |
| --- | --- |
| Age (years), mean (SD) (n_T_ =752) | 59.3(13.7) |
| BMI (kg/m²), median [IQR] (n_T_ =752) | 27.5 [23.0 ; 30.8] |
| Active Smokers (n_T_ =752) | 105 (14.0) |
| Diabetes (n_T_ =482) | 63 (13.1) |
| Menopausal status (n_T_ =433) |  |
| Menopause | 372 (85.9) |
| Perimenopause | 24 (5.5) |
| No Menopause | 37 (8.6) |
| Breast side (Right) (n_T_ =746) | 368 (49.3) |
| Quadrant (n_T_ =678) |  |
| Upper-Outer | 302 (44.5) |
| Overlapping lesion of breast | 109 (16.1) |
| Upper-Inner | 90 (13.3) |
| Central portion of breast | 70 (10.3) |
| Lower-Outer | 60 (8.8) |
| Lower-Inner | 43 (6.3) |
| Axillary tail of breast | 4 (0.6) |
| Molecular subtype (n_T_ =709) |  |
| Triple negative | 92 (13.0) |
| HR- HER2+ | 48 (6.8) |
| HR+ HER2+ | 67 (9.4) |
| HR+ HER2- | 502 (70.8) |
| Grade (n_T_ =689) |  |
| 1 | 110 (16.0 |
| 2 | 321 (46.6) |
| 3 | 258 (37.4) |
| pT stage (n_T_ =678) |  |
| 0 | 78 (11.6) |
| 1 | 238 (35.4) |
| 2 | 274 (40.7) |
| 3 | 67 (10.0) |
| 4 | 16 (2.4) |
| pN stage (n_T_ =694) |  |
| pN+ | 504 (72.6) |
| Breast/Chest Wall (n_T_ =752) |  |
| Breast | 415 (55.2) |
| Chest wall | 337 (44.8) |
| Axillary irradiation (n_T_ =752) | 1 (0.1) |
| Technique (n_T_ =752) |  |
| IMRT | 321 (42.7) |
| 3D | 431 (57.3) |
| Electrons (n_T_ =752) | 394 (52.4) |
| Boost (n_T_ =752) | 419 (55.7) |
| Photon Energy (n_T_ =752) |  |
| standard | 358 (47.6) |
| high | 394 (52.4) |
| Fractionation (n_T_ =752) |  |
| Normofractionated | 667 (88.7) |
| Hypofractionated | 85 (11.3) |
| BMI: Body Mass Index, IMRT: Intensity Modulated Radiation Therapy, IQR: InterQuartile Range, n_T_: number of patients with available information, pN: pathological Node (TNM classification), pT: pathological Tumor, S_diff_: Standardized Difference. SD: standard deviation, 3D: 3D conformal.  Results presented as n (%) unless otherwise specified. Percentages may not total 100 because of rounding. | |

Supplementary Table S4. Patient, tumor and treatment characteristics by fractionation group in patients with full data available for body mass index and smoking status

|  | Normofractionated (n=667) | Hypofractionated (n=85) | P value |
| --- | --- | --- | --- |
| Age (years), mean (SD) (n_T_ =752) | 58.2 (13.4) | 67.5 (13.9) | <0.0001 |
| BMI (kg/m²), median [IQR] (n_T_ =752) | 27.5 [23.1 ; 30.9] | 27.0 [23.1 ; 30.9] | 0.43 |
| Active Smokers (n_T_ =752) | 100 (15.0) | 5 (5.88) | 0.002 |
| Diabetes (n_T_ =482) | 52 (12.1) | 11 (20.37) | 0.09 |
| Menopausal status (n_T_ =433) |  |  | 0.06 |
| Menopause | 331 (84.7) | 41 (97.6) |  |
| Perimenopause | 23 (5.9) | 1 (2.4) |  |
| No Menopause | 37 (9.5) | 0 (0) |  |
| Breast side (Right) (n_T_ =746) | 326 (49.2) | 42 (50.6) | 0.22 |
| Quadrant (n_T_ =678) |  |  |  |
| Upper-Outer | 266 (44.2) | 36 (47.4) | 0.52 |
| Overlapping lesion of breast | 100 (16.6) | 9 (11.8) |  |
| Upper-Inner | 79 (13.1) | 12 (15.8) |  |
| Central portion of breast | 58 (9.6) | 12 (15.8) |  |
| Lower-Outer | 54 (9.0) | 6 (7.9) |  |
| Lower-Inner | 41 (6.8) | 2 (2.6) |  |
| Axillary tail of breast | 4 (0.8) | 0 (0) |  |
| Molecular subtype (n_T_ =709) |  |  | <0.0001 |
| Triple negative | 85 (13.4) | 7 (9.6) |  |
| HR- HER2+ | 44 (6.9) | 4 (5.5) |  |
| HR+ HER2+ | 52 (8.2) | 15 (20.5) |  |
| HR+ HER2- | 455 (71.5) | 47 (64.4) |  |
| Grade (n_T_ =689) |  |  | 0.047 |
| 1 | 91 (14.8) | 19 (25.3) |  |
| 2 | 287 (46.7) | 34 (45.3) |  |
| 3 | 236 (38.4) | 22 (29.3) |  |
| pT stage (n_T_ =673) |  |  | 0.11 |
| 0 | 74 (12.3) | 4 (5.5) |  |
| 1 | 218 (36.3) | 20 (27.4) |  |
| 2 | 237 (39.5) | 37 (50.7) |  |
| 3 | 58 (9.7) | 9 (12.3) |  |
| 4 | 13 (2.2) | 3 (4.1) |  |
| pN stage (n_T_ =694) |  |  | 0.13 |
| pN+ | 444 (71.7) | 60 (80.0) |  |
| Breast/Chest Wall (n_T_ =752) |  |  |  |
| Breast | 364 (54.6) | 51 (60.0) | 0.34 |
| Chest wall | 303 (45.4) | 34 (40.0) |  |
| Axillary irradiation (n_T_ =752) | 1 (0.1) | 0 (0) | 0.72 |
| Technique (n_T_ =752) |  |  |  |
| IMRT | 247 (37.0) | 84 (87.1) | <0.0001 |
| 3D | 420 (63.0) | 11 (12.9) |  |
| Electrons (n_T_ =752) | 386 (57.9) | 8 (9.4) | <0.0001 |
| Tumor bed boost (n_T_ =752) | 392 (53.2) | 64 (75.3) | 0.032 |
| Photon Energy (n_T_ =752) |  |  |  |
| Standard | 284 (42.6) | 74 (87.1) | <0.0001 |
| High | 383 (57.4) | 11 (12.9) |  |

BMI : Body Mass Index, HER2 : Human Epidermal Growth Factor 2, HR : hormone receptors, IMRT : Intensity Modulated Radiation Therapy, IQR: InterQuartile Range, n_T_: number of patients with available information, pN: pathological Node (TNM classification), pT: pathological Tumor, SD: standard deviation, 3D : 3D conformal.

Results presented as n (%) unless otherwise specified. Percentages may not total 100 because of rounding.

Supplementary Table S5. Patient, tumor and treatment characteristics according to occurrence of toxicity in patients with full data concerning BMI and smoking

|  | Acute toxicity (n=254) | No acute toxicity (n=498) | \|S_diff_\| % |
| --- | --- | --- | --- |
| Age (years), mean (SD) (n_T_ =752) | 57.73 (12.89) | 60.05 (14.10) | 17.1 |
| BMI (kg/m²), median [IQR] (n_T_ =752) | 28.01 [24.06 ; 32.21] | 25.84 [22.63 ; 30.22] | 34.4 |
| Active Smokers (n_T_ =752) | 46 (18.11) | 59 (11.85) | 17.6 |
| Diabetes (n_T_ =482) | 21 (12.73) | 42 (13.25) | 1.6 |
| Quadrant (n_T_ =678) |  |  |  |
| Lower Quadrant | 38 (16.10) | 65 (14.71) |  |
| Other | 198 (83.90) | 377 (85.29) | 3.9 |
| pT stage (n_T_ =673) |  |  |  |
| 0-1-2 | 220 (95.24) | 370 (83.71) | 38.24 |
| 3-4 | 11 (4.76) | 72 (16.29) |  |
| pN stage (n_T_ =694) |  |  |  |
| pN+ | 167 (71.67) | 337 (73.10) | 3.2 |
| Breast/Chest Wall (n_T_ =752) |  |  |  |
| Breast | 100 (36.76) | 257 (45.57) | 79.9 |
| Chest wall | 172 (63.24) | 307 (54.43) |  |
| Axillary irradiation (n_T_ =752) | 1 (0.39) | 0 (0) |  |
| Technique (n_T_ =752) |  |  |  |
| IMRT | 95 (37.40) | 226 (45.38) | 16.3 |
| 3D | 159 (62.60) | 272 (54.62) |  |
| Electrons (n_T_ =752) | 144 (56.69) | 250 (50.20) | 13.0 |
| Boost (n_T_ =752) | 198 (77.95) | 221 (44.38) | 73.4 |
| Photon Energy (n_T_ =752) |  |  |  |
| standard | 101 (39.76) | 257 (51.61) | 23.9 |
| high | 153 (60.24) | 257 (51.61) |  |

BMI: Body Mass Index, HER2: Human Epidermal Growth factor 2, HR: Hormone Receptors, IMRT: Intensity Modulated Radiation Therapy, IQR: InterQuartile Range, pN: pathological Node (TNM classification), n_T_: number of patients with available information, pT: pathological Tumor, S_diff_: Standardized Difference, SD: standard deviation 3D : 3D conformal.

Results presented as n (%) unless otherwise specified. Percentages may not total 100 because of rounding

Supplementary Table S6. Potential cofounders before and after propensity score weighting in patients with full data available for body mass index and smoking status

|  | Before weighting | | | After weighting | | | | |
| --- | --- | --- | --- | --- | --- | --- | --- | --- |
|  | Normo-fractionated (n=667) | Hypo-fractionated (n=85) | \|S_diff_\|  % | Normo-fractionated (n=1414) | Hypo-fractionated (n=307) | \|S_diff_\|  % | % bias reduction | P value |
| Age (years), mean (SD) | 58.22 (13.38) | 67.46 (13.89) | 67.8 | 60.08 (14.08) | 61.06(53.84) | 7.2 | 89.4 | 0.42 |
| BMI (kg/m²), median [IQR] | 27.52  [23.14 ; 30.95] | 26.96  [23.14 ; 30.95] | 9.3 | 26.75  [23.42 ; 26.75] | 25.03  [23.63 ; 27.63] | 12.9 | 0 | <0.0001 |
| Active smokers | 14.99 (100) | 5.88 (5) | 30.1 | 11.11 | 4.66 | 21.3 | 29.2 | <0.0001 |
| Breast/chest wall |  |  |  |  |  |  |  | 0.06 |
| Breast | 54.57 (364) | 60.00 (51) | 11.0 | 56.20 | 61.15 | 10.0 | 9.1 |  |
| Chest wall | 45.43 (303) | 40.00 (34) |  | 43.80 | 38.85 |  |  |  |
| IMRT | 37.03 (247) | 87.06 (84) | 120.0 | 45.02 | 46.33 | 3.1 | 97.4 | 0.62 |
| Electrons | 57.87 (386) | 9.41 (8) | 119.5 | 49.82 | 49.96 | 8.0 | 93.3 | 0.20 |
| Boost | 53.19 (392) | 64.65 (64) | 12.5 | 56.86 | 63.12 | 12.7 | 0 | 0.02 |
| Energy |  |  | 105.3 |  |  | 8.0 | 92.4 | 0.06 |
| Standard | 42.58 (284) | 87.06 (74) |  | 48.42 | 51.82 |  |  |  |
| High | 57.42 (383) | 12.94 (11) |  | 51.58 | 48.18 |  |  |  |

BMI: Body Mass Index, IMRT: Intensity Modulated Radiation Therapy, IQR: InterQuartile Range, S_diff_: Standardized Difference, SD: standard deviation.

Results presented as n (%) unless otherwise specified. Percentages may not total 100 because of rounding.

Supplementary Table S7. Sensitivity analyses results after IPTW and adjustment for imbalanced baseline parameters after weighting.

|  | OR, 95% CI * |
| --- | --- |
| Fractionation group | 0.10 [0.07;0.14] |
| Smoking | 1.53 [0.99;2.36] |
| Boost | 5.42 [3.88;7.59] |

*Weighted on propensity score

OR, odds ratio; CI, confidence interval.

Supplementary Figure 1. Plot of standardized differences before and after weighting
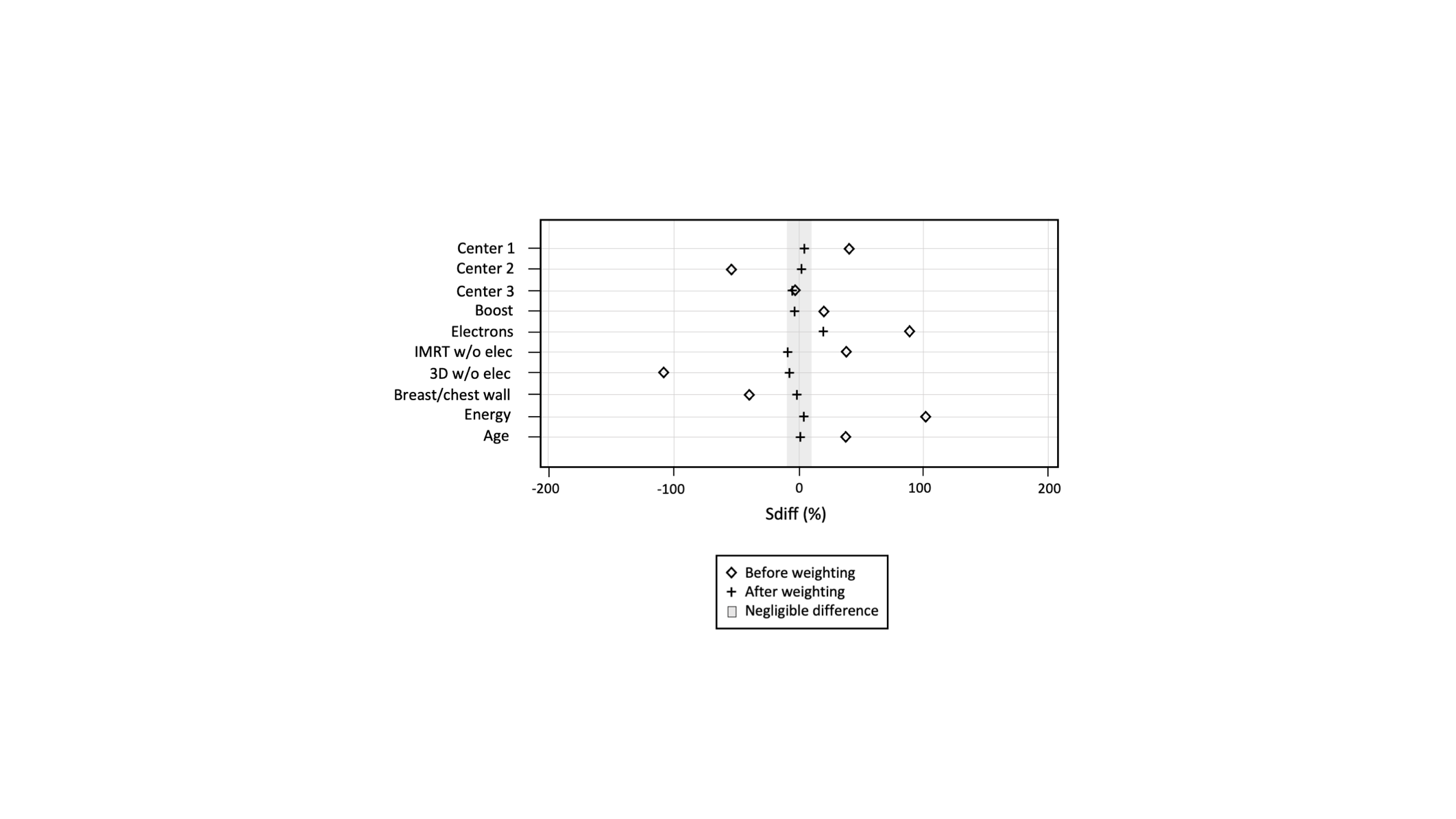


IMRT: Intensity Modulated Radiation Therapy, 3D: 3D conformal, w/o elec : without electrons

Supplementary Figure 2. Plot of standardized differences before and after weighting for sensitivity analysis


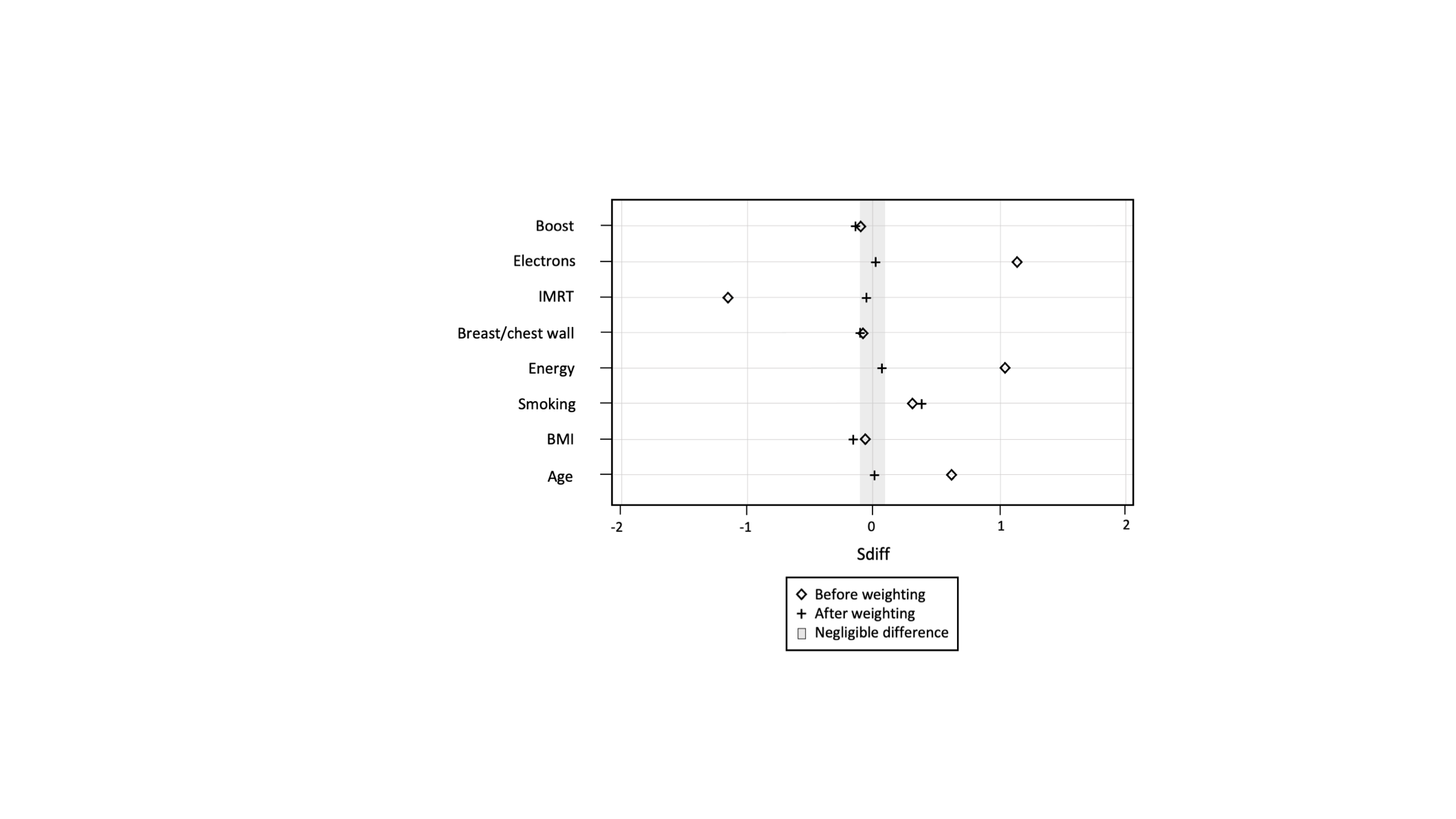


IMRT: Intensity Modulated Radiation Therapy, BMI: Body Mass Index

Supplementary Figure 3. Proportion of hypofractionated radiotherapy over time.


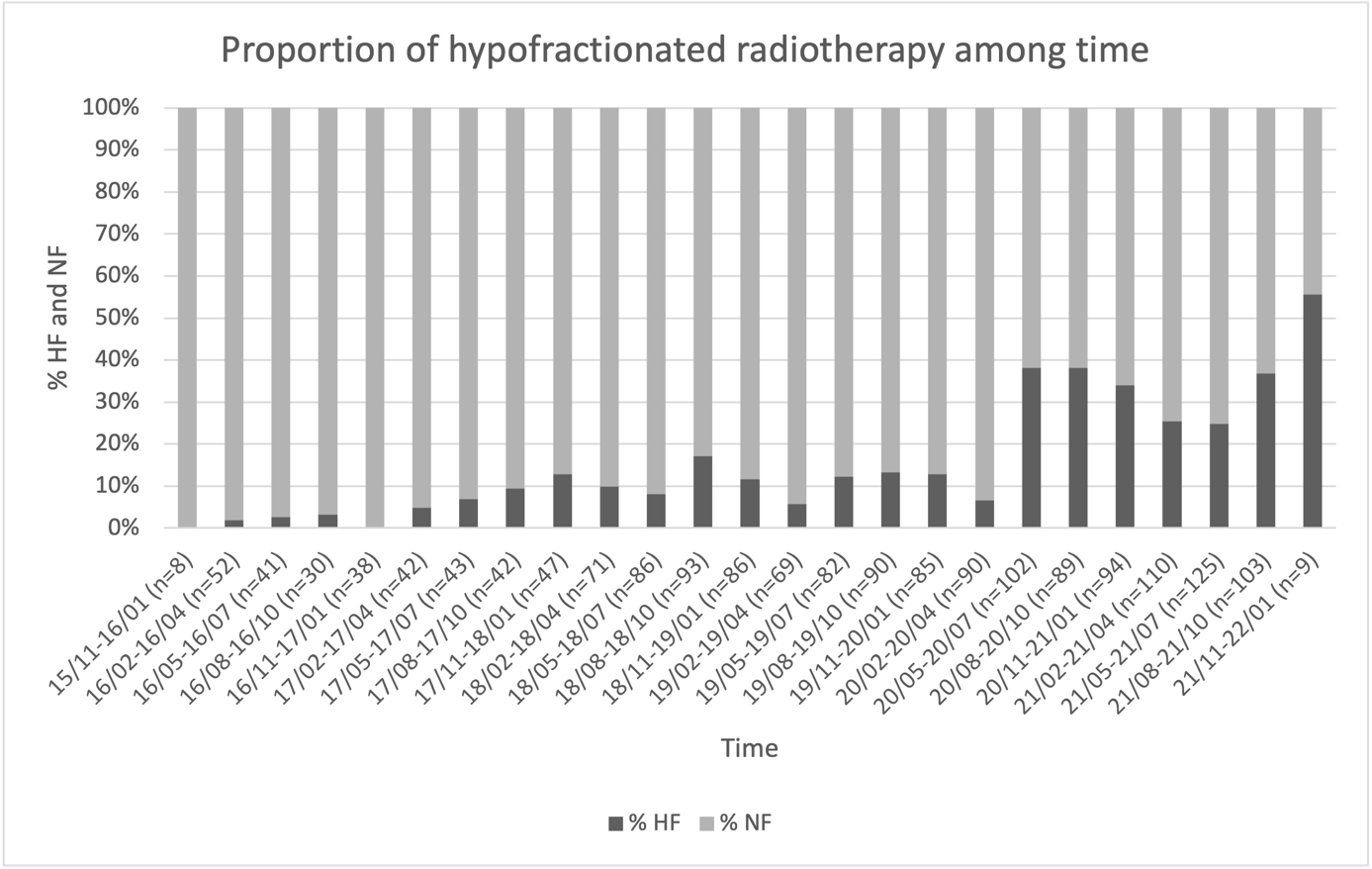


HF: Hypofractionated, NF : Normofractionated.
